# Comparison of ^18^F-DCFPyL and ^68^Ga-PSMA-11 for ^177^Lu-PSMA-617 therapy patient selection

**DOI:** 10.3389/fonc.2024.1382582

**Published:** 2024-06-27

**Authors:** Surekha Yadav, Sarasa T. Kim, Abuzar Moradi Tuchayi, Fei Jiang, Amanda Morley, Rachelle Saelee, Yingbing Wang, Roxanna Juarez, Courtney Lawnh-Heath, Vadim S. Koshkin, Thomas A. Hope

**Affiliations:** ^1^ Department of Radiology and Biomedical Imaging, University of California, San Francisco, San Francisco, CA, United States; ^2^ Department of Epidemiology & Biostatistics, University of California, San Francisco, San Francisco, CA, United States; ^3^ Division of Hematology/Oncology, Department of Medicine, University of California, San Francisco, San Francisco, CA, United States; ^4^ Helen Diller Family Comprehensive Cancer Center, University of California, San Francisco, San Francisco, CA, United States; ^5^ Department of Radiology, San Francisco Veterans Affairs (VA) Medical Center, San Francisco, CA, United States

**Keywords:** ^68^Ga-PSMA PET/CT, ^18^F-DCFPyL-PET/CT, ^177^Lu-PSMA, radioligand therapy (RLT), mCRPC, patient screening

## Abstract

**Purpose:**

^68^Ga-PSMA-11 is recommended for the selection of patients for treatment in the package insert for ^177^Lu-PSMA-617. We aimed to compare imaging properties and post-treatment outcomes from radioligand therapy (RLT) of patients selected with ^68^Ga-PSMA-11 and ^18^F-DCFPyL.

**Methods:**

We retrospectively evaluated 80 patients undergoing PSMA RLT, who had pretreatment imaging using either ^68^Ga-PSMA-11 or ^18^F-DCFPyL. For both groups, we compared the biodistribution and lesion uptake and the PSA response to treatment.

**Results:**

Both agents had comparable biodistribution. Patients initially imaged with ^18^F-DCFPyL had a higher PSA response (66% vs. 42%), and more patients had a PSA50 response (72% vs. 43%) compared to patients imaged with ^68^Ga-PSMA-11.

**Conclusion:**

^18^F-DCFPyL and ^68^Ga-PSMA-11 had comparable biodistribution and lesion uptake. Patients imaged with ^18^F-DCFPyL demonstrated clinical benefit to PSMA RLT comparable to those imaged with ^68^Ga-PSMA-11, and either agent can be used for screening patients.

## Introduction

^177^Lu-PSMA-617 radioligand therapy (RLT) has been shown to improve clinical outcomes and have a favorable safety profile in men with metastatic castration resistant prostate cancer (mCRPC) (1–5). In the phase 3 VISION study, ^177^Lu-PSMA-617 RLT was shown to prolong overall survival (OS) and improve quality of life measures in patients with mCRPC relative to best supportive care (3), while the Phase 2 TheraP Trial demonstrated that ^177^Lu-PSMA-617 resulted in a higher rate of PSA decline relative to cabazitaxel chemotherapy (4). When compared to conventional imaging, prostate-specific membrane antigen (PSMA)-based positron emission tomography (PET) has higher detection rates and greater diagnostic accuracy for patients with initial high risk, biochemically recurrent or persistent prostate cancer, and mCRPC (6–9). In this theranostic approach, PSMA PET is used for the screening of patients to demonstrate the presence of PSMA expression, which makes them eligible for PSMA RLT (10–12).

There are three FDA-approved PSMA ligands for PET imaging: ^68^Ga-PSMA-11 (gozetotide), ^18^F-DCFPyL (piflufolostat), and rhPSMA-7.3 (posluma). A series of phase III trials have evaluated the use of ^68^Ga-PSMA-11, ^18^F-DCFPyL-PET/CT, and rhPSMA-7.3 in prostate cancer patients at initial staging and biochemical recurrence (7–9, 13–15). Most consider the diagnostic utility of the three PSMA ligands to be equivalent at initial staging and biochemical recurrence. VISION and TheraP trials used ^68^Ga-PSMA-11 PET for their trials due to the extensive clinical experience and wide availability (3, 4). Screen failures were later shown to be associated with poorer outcomes (16).

Despite the market availability of ^18^F-DCFPyL, the ^177^Lu-PSMA-617 (vipivotide tetraxetan) package insert specifically recommends selecting patients for treatment using ^68^Ga-PSMA-11, as the imaging agent and the utility of other PSMA ligands to select patients remains unclear. We retrospectively evaluated patient outcomes and imaging properties of ^68^Ga-PSMA-11 and ^18^F-DCFPyL in patients undergoing PSMA RLT in order to help determine if ^18^F-DCFPyL is appropriate to use for patient selection.

## Material and methods

### Study population

In this study, we retrospectively screened individuals who underwent pre-treatment PET imaging with either ^68^Ga-PSMA-11 or ^18^F-DCFPyL before ^177^Lu-PSMA-617 RLT at our institution from October 2021 to April 2023. The selection of radiopharmaceutical was determined by availability at each imaging center. Included patients had PSMA PET performed within 6 months prior to the first cycle of ^177^Lu-PSMA-617 RLT. This study was approved by the institutional review board, and informed consent was waived.

### PSMA PET acquisition

Patient preparation and administration of either of the PSMA ligands was done as per standard published guidelines (11). The median injected activity of ^68^Ga-PSMA-11 was 5.7 mCi (4.9–11.4). The median injected activity of ^18^F-DCFPyL was 9.8 mCi (7.0–11.7). Median uptake time was 58 min (50–102) for ^68^Ga-PSMA-11 and 60 min (52–101) for ^18^F-DCFPyL, respectively. A vertex to mid-thigh PET scan was performed using either PET/CT or PET/MRI.

### Image interpretation

Each PSMA PET scan was interpreted using Visage (Visage Imaging). Five regions were recorded for the presence of prostate cancer including prostate bed (T), osseous (M1b), pelvic nodes (N), extrapelvic nodes (M1a), and visceral metastases (M1c). Maximum standardized uptake values (SUVmax) were recorded for osseous metastases, extrapelvic nodes, and visceral metastases with the highest uptake. Additionally, SUV was also recorded for physiological uptake in the liver (SUVmean) and parotid glands (SUVmax).

### Response to RLT

Serum PSA levels served as the standard of reference for response assessment to ^177^Lu-PSMA-617 RLT (17–19). The maximum decline in PSA that occurred anytime during or within 12 weeks of completion of RLT was taken for PSA response analysis. Baseline serum PSAs were drawn on the day of cycle 1 of RLT treatment, and the best PSA response during RLT was assessed for each patient. A decline of 50% from baseline PSA was defined as PSA50 response.

### Statistical plan

Descriptive statistics in the form of median (interquartile) for continuous variable and count (percentage) for the binary variables was used to describe quantitative variables from the clinical data. While comparing the baseline characteristics between the groups imaged with ^68^Ga-PSMA-11 and ^18^F-DCFPyL; Student’s t-test was used for the continuous variables and Fisher exact test was used for the discrete variables. A Student’s t-test was conducted to assess the relationship between the lesion SUV and organ uptake between the groups imaged with ^68^Ga-PSMA-11 and ^18^F-DCFPyL. For the comparison of SUV and response, the median SUVmax was used to split the population evenly. p <0.05 was considered significant. A comparison of the best overall post-treatment PSA relative to baseline PSA was made between ^68^Ga-PSMA-11 and ^18^F-DCFPyL using Student’s t-test. The maximum decline in PSA during RLT was reported for each patient using waterfall plots.

## Results

### Patient characteristics

Of the 80 patients who received ^177^Lu-PSMA-617 therapy at our institution from June 2022 to June 2023, 47 patients received ^68^Ga-PSMA-11 and 33 patients received ^18^F-DCFPyL for pre-treatment PET imaging. The patients in both these groups were similar for age, Gleason score, and pre-therapy PSA levels, and prior treatments. Patients imaged using ^68^Ga-PSMA-11 had higher rates of prior radiation therapy. The two groups had a similar distribution of disease in the prostate/prostate bed, and metastatic disease to lymph nodes (N1), bone (M1a), soft tissue (M1b), and distant organs (M1c) (**Table 1**).

**Table 1:** Patient demographics.

	^68^Ga-PSMA-11	^18^F-DCFPyL	Overall	p-value
Patients, n	47	33	80	Not available
Age, median (IQ)	74 (68, 80)	72 (66, 78)	72 (67, 79.25)	0.36
Gleason grade group median (IQ)	5 (4, 5)	4 (3, 5)	4 (4, 5)	0.93
PSA at baseline median (IQ)	11.8 (6.4, 74.63)	27 (6, 103)	22.35 (6.27, 92.72)	0.39
Prior Treatments (n, %)				
ADT, n (%)	47 (100)	33 (100)	80 (100)	1
ARTT, n (%)	47 (100)	33 (100)	80 (100)	1
Chemotherapy, n (%)	46 (98)	33 (100)	79 (99)	1
Radical prostectomy, n (%)	17 (36)	8 (24)	25 (31)	0.33
Radiation therapy, n (%)	40 (85)	22 (67)	62 (78)	0.06
Site of disease				
Prostate bed, n (%)	14 (30)	9 (27)	23 (29)	1
Bone, n (%)	46 (98)	31 (94)	77 (96)	0.57
Lymph node, n (%)	33 (70)	24 (73)	57 (71)	1
Visceral, n (%)	18 (38)	11 (33)	29 (36)	0.81
Pre-therapy PSA, median (IQ)	134.45 (42.58, 300.5)	209.18 (36.76, 1307.99)	134.45 (37.14, 465.36)	0.22
Post-therapy nadir PSA, median (IQ)	51.9 (4.33, 150.79)	32.72 (8.19, 419.27)	36.71 (7.75, 204.68)	0.27
Administered activity (mCi) (median, range)	5.72 (5.3, 6.4)	9.82 (9.38, 10.27)	6.6 (5.58, 9.6)	0
Time to imaging (mins) (median, IQ)	58 (54.25, 66)	60 (57.5, 64)	60 (55, 66)	0.66
Interval between imaging and RLT (days) (median, IQ)	72 (54–106)	64 (41–77)	67 (42–95)	0.26
Median number of RLT cycles (IQ)	4 (2, 5)	4 (3, 6)	4 (2, 6)	0.25

ADT, androgen deprivation therapy; ARTT, androgen receptor–targeted therapy.

### Physiological biodistribution

No statistically significant difference was observed between the two groups for the liver SUVmean (4.2 ± 1.7 for ^68^Ga-PSMA-11 versus 4.2 ± 1.5 for ^18^F-DCFPyL, p=0.99) or the parotid SUVmax (15.2 ± 5.4 for ^68^Ga-PSMA-11 versus 14.5 ± 7.3 for ^18^F-DCFPyL, p=65; **Table 2**).

**Table 2:** Physiological biodistribution and metastatic lesion parameters for ^68^Ga-PSMA-11 and ^18^F-DCFPyL.

Site	^68^Ga-PSMA-11 Median SUV (IQR)	^18^F-DCFPyL Median SUV (IQR)	p-value	Overall Median SUV (IQR)
Physiological biodistribution				
Parotid glands SUV max	15.2 (± 5.4)	14.5 (± 7.3)	0.65	14.9 (± 6.2)
Liver SUV mean	4.2 (± 1.7)	4.3 (± 1.5)	0.99	4.2 (± 1.6)
Metastatic lesions				
Extrapelvic lymph nodes	28.9 (11.1–37.1)	29.7 (14.1–43.5)	0.33	29.5 (12.6–40.4)
Osseous	31.7 (15.0–56.9)	30.0 (25.3–58.7)	0.39	30.6 (21.0–58.2)
Visceral	11.2 (9.0–20.9)	28.7 (23.9–32.2)	0.04	18.8 (9.5–28.5)
Most PSMA avid lesion	34.4 (19.6–60.7)	34.0 (26.8–60.8)	0.79	34.2 (23.0–61.4)

### Radiopharmaceutical and PSA response analysis

**Figure 1:**
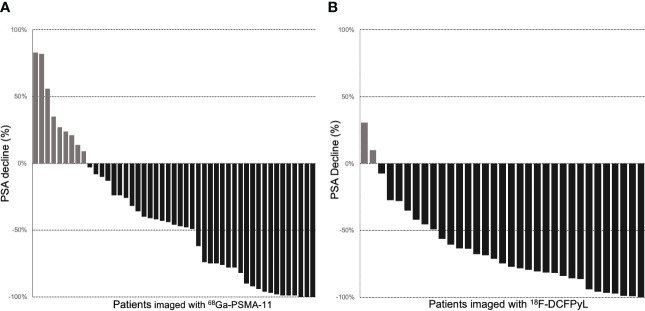
Waterfall plots of PSA response to RLT in patients with pretreatment PET imaging with ^68^Ga-PSMA-11 **(A)** and ^18^F-DCFPyL **(B)**.

**Figure 2:**
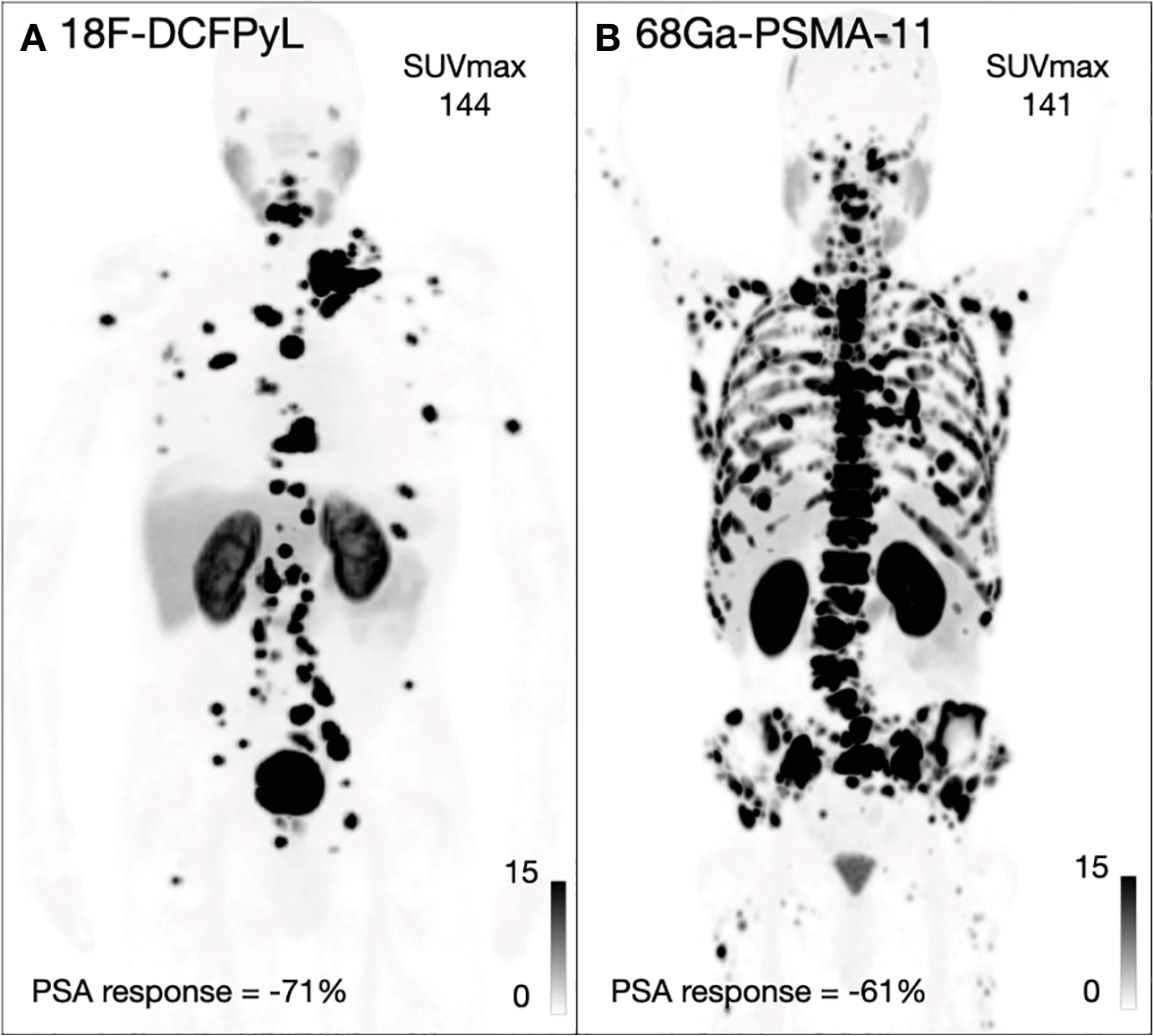
Two example patients imaged with 18F-DCFPyL **(A)** and 68Ga-PSMA-11 **(B)** including the SUVmax for both patients and maximum PSA response.

Among 47 patients imaged with ^68^Ga-PSMA-11, 38 (80%) patients had PSA decrease relative to baseline. The average PSA response from baseline was 42%, and 20 (43%) patients had a >50% reduction in PSA (PSA50). Among 33 patients imaged with ^18^F-DCFPyL, 31 (93%) patients had PSA decrease relative to baseline (**Figure 1**). The average PSA response from baseline was 65%, and 24 (72%) patients had a PSA50 response. The PSA50 response was higher for patients imaged with ^18^F-DCFPyL prior to treatment compared to ^68^Ga-PSMA-11 (p-value = 0.03; **Supplementary Table S1**, **Figure 2**).

### Semi-quantitative comparison of metastatic lesions on pretreatment PET imaging

In comparing the highest SUVmax lesion for the three metastatic sites between the ^68^Ga-PSMA-11 and ^18^F-DCFPyL groups, no statistically significant difference was observed between the extrapelvic lymph nodes (p=0.33) or osseous lesions (p=0.39), respectively. For visceral metastatic lesions, ^18^F-DCFPyL had a higher uptake than ^68^Ga-PSMA-11 (median = 11.2 (9.0–20.9) for ^68^Ga-PSMA-11 = versus median = 28.7 (23.9–32.2) for ^18^F-DCFPyL, p=0.04; **Table 2**).

The median SUVmax across the overall population was 34.2 and was used to divide patients into two groups: those with high uptake (SUVmax > 34.2) and those with low uptake (SUVmax < 34.2). There was a trend to a higher PSA response in patients imaged with ^18^F-DCFPyL compared to ^68^Ga-PSMA-11, which was not statistically significant (**Supplementary Table S2**).

## Discussion

This is the first report of outcomes in patients treated with PSMA RLT, who were selected with ^18^F-DCFPyL. We demonstrated that the biodistribution between the two agents was identical and showed that the PSA50 response in patients selected with ^18^F-DCFPyL was higher than in patients selected for treatment with ^68^Ga-PSMA-11. Although prior work has focused on the diagnostic utility of ^18^F-DCFPyL, our results demonstrate that it is appropriate to use it for PSMA RLT screening.

The PSA response to RLT was higher in patients who underwent pretreatment PET imaging with ^18^F-DCFPyL than those with ^68^Ga-PSMA-11. This was unexpected, and it is unclear from our small patient numbers if this finding is generalizable. Overall, our results indicate that patients selected with ^18^F-DCFPyL appear to benefit at least equally to PSMA RLT, which is consistent with guidelines that indicate that either agent can be used for patient selection (11, 12).

As has been previously reported, the biodistributions were similar between the two agents (20, 21). The SUVmax of metastatic lesions are comparable for extrapelvic lymph nodes and osseous sites. While ^18^F-DCFPyL outperforms ^68^Ga-PSMA-11 in having higher uptake in visceral lesions, this is limited by the number of lesions included, and intra-patient comparison is needed to confirm that there is in fact higher uptake in visceral lesions.

This work has focused on the difference between ^18^F-DCFPyL and ^68^Ga-PSMA-11, but with the recent approval of rhPSMA-7.3, it is uncertain how our results can be extrapolated to include this newer radiopharmaceutical, especially given the partial hepatobiliary clearance seen with rhPSMA-7.3. Although rhPSMA-7.3 has been shown to have lower urinary excretion, which may lead to enhance visualization of local recurrence, the liver uptake is higher than in the other two agents. At this time, it is unclear what the threshold should be for patient selection when using rhPSMA-7.3.

Our study has several limitations. First, this was a retrospective study. This study is additionally subject to various confounders inherent to its lack of intra-patient comparison of the two imaging agents. Specifically, mCRPC exhibits significant heterogeneity, and our analysis only considers the lesion with highest avidity, which may not fully represent the disease burden. The ^18^F-DCFPyL group received more treatment cycles, and their visceral metastatic lesions showed higher radiotracer avidity, which would be expected to impact the PSA response rate. In the absence of head-to-head trials, comparisons of reported radiopharmaceutical performance on outcomes should be interpreted with caution due to the significant impact of differing patient populations, treatment cycles, end points, scanning protocols, scanning equipment, and readers.

## Conclusion

Patients imaged with ^18^F-DCFPyL demonstrated clinical benefit to PSMA RLT in this retrospective study. As previously shown, the physiological biodistribution and lesion uptakes at metastatic sites are comparable for both agents. Patients selected with ^18^F-DCFPyL had higher PSA50 responses, but comparison to ^68^Ga-PSMA-11 is limited given the many confounders. Although ^68^Ga-PSMA-11 was utilized for patient selection in clinical trials of ^177^Lu-PSMA-617, screening patients can be done using either of radiopharmaceuticals and should not be limited to ^68^Ga-PSMA-11.

## Data availability statement

The original contributions presented in the study are included in the article/**Supplementary Material**. Further inquiries can be directed to the corresponding author.

## Ethics statement

The studies involving humans were approved by institutional review board, University of California San Francisco. The studies were conducted in accordance with the local legislation and institutional requirements. Written informed consent for participation was waived off from the participants or the participants’ legal guardians/next of kin in accordance with the institutional requirements.

## Author contributions

SY: Writing – review & editing, Writing – original draft, Software, Project administration, Methodology, Investigation. SK: Writing – review & editing, Methodology, Investigation, Data curation. AT: Writing – review & editing, Project administration, Methodology, Data curation. FJ: Writing – review & editing, Validation, Software, Formal analysis. AM: Writing – review & editing, Project administration, Investigation, Data curation. RS: Writing – review & editing, Project administration, Investigation, Data curation. YW: Writing – review & editing, Validation, Supervision, Formal analysis. RJ: Writing – review & editing, Validation, Supervision, Formal analysis. CL: Writing – review & editing, Validation, Supervision, Formal analysis. VK: Writing – review & editing, Validation, Supervision, Formal analysis. TH: Writing – review & editing, Visualization, Validation, Supervision, Resources, Project administration, Methodology, Funding acquisition, Formal analysis, Conceptualization.
