# Supplementary material for: Comparison of 18F-DCFPyL and 68Ga-PSMA-11 for 177Lu-PSMA-617 therapy patient selection
Source: Front Oncol. 2024 Jun 27;14:1382582. doi: 10.3389/fonc.2024.1382582 (PMC11238039; doi:10.3389/fonc.2024.1382582)
Supplement: Supplementary file 1 [file Table_1.pdf]

Supplemental files:

Supplemental Table 1: PSA response for groups imaged with ^{68}Ga-PSMA-11 and ^{18}F-DCFPyL

Number of RLT cases	^{68}Ga-PSMA-11 N=47, Avg PSA response = 42%	^{18}F-DCFPyL N=33, Avg PSA response = 65%	P value
Any PSA response	38 (80%)	31 (93%)	0.26
Average PSA response	26 (55%)	20 (60%)	0.22
PSA50 response	20 (43%)	24 (72%)	0.03

Supplemental Table 2: SUV parameter impact on PSA response for PSMA-11 and DCFPyL, stratified by median parotid SUVmax of 14, and median metastases SUVmax of 34.2. *p = 0.02

	Average PSA response (%)		
Parotid SUVmax	PSMA-11	DCFPyL	Overall % (n=39)
>14	34.1, n=24	61.3, n=15	44.5, n=39
<14	41.4, n=23	62.3, n-=18	53.1, n=41
Lesion SUVmax	Metastatic lesions		
Extrapelvic Lymph nodes			

>34.2	82.2, n=11	64.7, n=8	74.8, n=19
<34.2 *	10.0, n=18	54.6, n=11	27, n=29
Osseous lesions			
>34.2	51.5, n=21	70.1, n=13	58.6, n=34
<34.2	34.3, n=25	57.2, n=17	43.6, n=42
Visceral lesions			
>34.2	88.4, n=2	24.9, n=2	56.6, n=4
<34.2	43.5, n=16	50.8, n=8	45.9, n=24
Most PSMA avid lesions			
>34.2	55.4, n=24	71.0, n=17	61.6, n=40
<34.2	27, n=23	51.6, n=16	37.0, n=39
